# Secreted Ligands of the NK Cell Receptor NKp30: B7-H6 Is in Contrast to BAG6 Only Marginally Released via Extracellular Vesicles

**DOI:** 10.3390/ijms22042189

**Published:** 2021-02-22

**Authors:** Viviane Ponath, Nathalie Hoffmann, Leonie Bergmann, Christina Mäder, Bilal Alashkar Alhamwe, Christian Preußer, Elke Pogge von Strandmann

**Affiliations:** Institute for Tumor Immunology, Clinic for Hematology, Immunology and Oncology, Philipps University of Marburg, Hans-Meerwein-Strasse 3, 35043 Marburg, Germany; ponath@staff.uni-marburg.de (V.P.); nathalie-Hoffmann@web.de (N.H.); leonie.bergmann@uke.de (L.B.); maeder.christina@gmx.de (C.M.); alashkab@staff.uni-marburg.de (B.A.A.); christian.preusser@staff.uni-marburg.de (C.P.)

**Keywords:** extracellular ligands, BAG6, B7-H6, NKp30, tumor cell killing

## Abstract

NKp30 (Natural Cytotoxicity Receptor 1, NCR1) is a powerful cytotoxicity receptor expressed on natural killer (NK) cells which is involved in tumor cell killing and the regulation of antitumor immune responses. Ligands for NKp30, including BAG6 and B7-H6, are upregulated in virus-infected and tumor cells but rarely detectable on healthy cells. These ligands are released by tumor cells as part of the cellular secretome and interfere with NK cell activity. BAG6 is secreted via the exosomal pathway, and BAG6-positive extracellular vesicles (EV-BAG6) trigger NK cell cytotoxicity and cytokine release, whereas the soluble protein diminishes NK cell activity. However, the extracellular format and activity of B7-H6 remain elusive. Here, we used HEK293 as a model cell line to produce recombinant ligands and to study their impact on NK cell activity. Using this system, we demonstrate that soluble B7-H6 (sB7-H6), like soluble BAG6 (sBAG6), inhibits NK cell-mediated target cell killing. This was associated with a diminished cell surface expression of NKG2D and NCRs (NKp30, NKp40, and NKp46). Strikingly, a reduced *NKp30* mRNA expression was observed exclusively in response to sBAG6. Of note, B7-H6 was marginally released in association with EVs, and EVs collected from B7-H6 expressing cells did not stimulate NK cell-mediated killing. The molecular analysis of EVs on a single EV level using nano flow cytometry (NanoFCM) revealed a similar distribution of vesicle-associated tetraspanins within EVs purified from wildtype, BAG6, or B7-H6 overexpressing cells. NKp30 is a promising therapeutic target to overcome NK cell immune evasion in cancer patients, and it is important to unravel how extracellular NKp30 ligands inhibit NK cell functions.

## 1. Introduction

Clinical and experimental data in different model systems and tumor entities demonstrate the important role of natural killer (NK) cells in the immunological control of tumors [1,2,3]. The regulation of the direct cytotoxicity of NK cells is based on the interaction of inhibitory receptors, which interact with MHC class I encoded self-antigens on the target cells, and activating receptors. The major activating receptors of NK cells are FcRIIIa (CD16a), DNAM, NKG2D, and the group of NCRs with NKp30, NKp44, and NKp46. These receptors are stimulated by stress-inducible ligands that are expressed on virus-infected or malignant cells but are largely absent on healthy cells [4,5,6]. Activation leads to direct lysis of the target cells via death receptors and the release of cytotoxic granules. In addition, inflammatory cytokines such as IFNγ or TNFα are secreted, and thus, NK cells also contribute to the adaptive immune response [7,8] and shape the tumor microenvironment, e.g., in gastrointestinal or pancreatic tumors [9,10].

However, ligands for activating NK cell receptors are also released by tumor cells and part of the cellular secretome. These extracellular ligands may interfere with NK cell regulation and antitumor immune responses, representing an important immune escape mechanism. Consequently, ligands are then no longer presented on the surface of the tumor cells, thus preventing recognition by NK cells. Furthermore, chemokines or soluble ligands can inhibit the activation of NK cells via downregulation of cytotoxic receptors [11,12,13,14,15,16,17]. The NKG2D/NKG2D ligand system is very well studied in this context, and recently, it has been shown that antibodies which prevent shedding of NKG2D ligands inhibit tumor growth in multiple mouse models [18,19]. Targeting the NKG2D/NKD2D ligand axis is a promising approach in novel immunotherapies [20,21,22].

In contrast to NKG2D ligands, the role and activity of NKp30 ligands BAG6 and B7-H6, either cell-surface expressed or present in the cellular secretome, are not well understood. A study with patients with gastrointestinal (GIST) tumors provides the first indication of a connection of a high sBAG6 level associated with a low NKp30 level and of their clinical relevance. Here, sBAG6 levels correlate with a low NKp30 expression, and sBAG6 is a predictive biomarker for the response to imatinib and the survival of the patients [23,24]. In the GIST study, high serum levels of B7-H6 were also detected and identified as a predictive marker. Additionally, in patients with metastatic neuroblastoma, the expression of NKp30 isoforms correlates with NK cell activity and survival [25]. Some of the patients in this study had high serum levels of the soluble NKp30 ligand B7-H6, which were associated with reduced NK cell activity and continued to be associated with chemoresistance and survival.

In contrast to B7-H6 [26], the NKp30 ligand BAG6 [27] is not a classical surface molecule but a nuclear protein, which is released upon stress signals via extracellular vesicles (EVs) from tumor and dendritic cells. EVs presenting BAG6 (EV-BAG6) activate NK cells via the NKp30–BAG6 axis [27,28]; however, BAG6-positive vesicles were hardly detectable in the serum of tumor patients. In contrast, high levels of sBAG6 were detectable in patients correlating with the stage of the disease [16,17]. Of note, sBAG6 inhibited the cytotoxicity of NK cells and the secretion of the proinflammatory cytokines TNFα/IFNγ in line with a general decrease of activating receptors on NK cells after contact with sBAG6-positive patient sera. The role of B7-H6 in vesicle-associated NK cell regulation has not been addressed so far.

Here, we purified vesicles as well as recombinant soluble ligands from cells overexpressing BAG6 and B7-H6, respectively, to characterize the different ligand natures and analyze their impact on NK cell activity.

## 2. Results and Discussion

**Figure 1:**
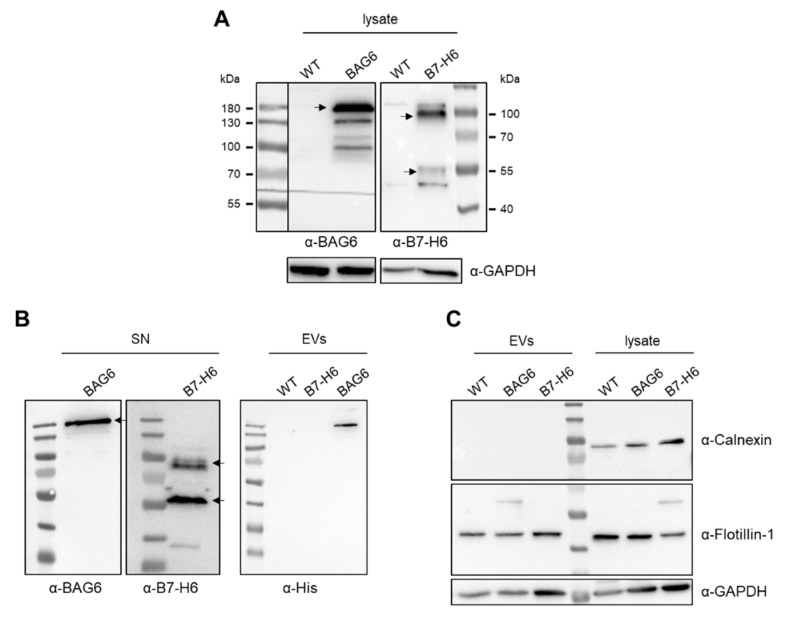
Detection of extracellular vesicle (EV)-related and overexpressed proteins after transfection. (**A**) Western blot analysis of overexpressed BAG6 (left) and B7-H6 (right) in HEK293 cells after 48 h of transfection. (**B**) BAG6 and B7-H6 were released into the supernatant (left), and BAG6 was detectable in EV lysates (right) 48 h after transfection. (**C**) Flotillin-1 was detected as an EV marker, and Calnexin was used as a negative control. WT = wildtype, SN = supernatant.

BAG6 and B7-H6 overexpression vectors were generated and the recombinant proteins were detectable in the cell lysates of transfected cells (Figure 1A). Both ligands were released by HEK293 cells, and the soluble proteins were detectable in the cell supernatant (Figure 1B). The BAG6 signal was detected at approximately 180 kDaand B7-H6 at 80 kDa and 50 kDa corresponded to the glycosylated full-length protein and a truncated form, respectively, which is known to be released from the cell surface upon metalloprotease shedding [14]. Moreover, the recombinant BAG6 protein was detected on EVs purified from the cell supernatant of BAG6-transfected cells. In contrast, B7-H6 was not detectable in EV samples from B7-H6 transfected cells, suggesting that this ligand did not or only marginally released in a vesicle-associated form (Figure 1B, right panel). Excluding major cellular contamination, we could not detect calnexin in the EV samples, whereas vesicle-associated molecules, including flotillin-1 and glyceraldehyde-3-phosphate dehydrogenase (GAPDH), were present (Figure 1C). Thus, Western blot data suggest that B7-H6 was, in contrast to BAG6, not released in association with vesicles.

**Figure 2:**
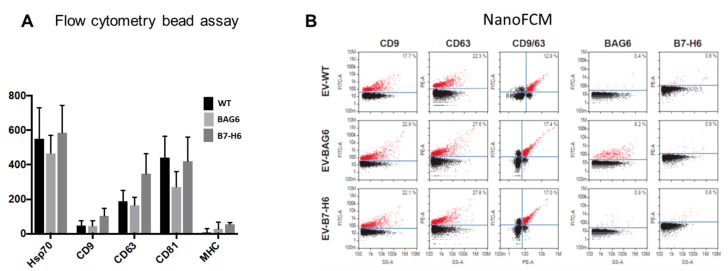
Detection of EV markers on the surface of EVs. (**A**) The tetraspanins CD9, CD63, and CD81, as well as Hsp70 and MHC-1, were detected on EVs using flow cytometry bead assay. Data are the mean of four to eight experiments ± SEM. (**B**) Representative plots of CD9, CD63, BAG6, and B7H6 expression on single EVs using FITC and PE-conjugated antibodies by nano flow cytometry (NanoFCM analysis (*n* = 3)). Bivariate dot-plots of indicated fluorescence versus side scatter (SS-A) are shown. In addition, double positives for CD9/CD63 are depicted.

To further characterize vesicles on a molecular level, we applied bead-based flow cytometry and nano flow cytometry (NanoFCM) analysis to perform single vesicle phenotyping. As expected, vesicle markers including Hsp70; tetraspanins CD9, CD63, and CD81; and low levels of MHC were detected using bead assays, and the individual vesicle samples show only minor differences (Figure 2A). This method is often applied to validate that purified particle/vesicle fractions represent in fact EVs. In contrast to conventional flow cytometry, NanoFCM allows the characterization of individual particles, as the resolution limit is reduced to 40 nm. Thus, small EVs (40–200 nm) can be analyzed in concentration and size in addition to fluorescence-based marker detection. Interestingly, these data revealed that about 20% of all particles expressed CD9 or CD63, in which most of them were double-positive for both tetraspanins. In line with the Western blot analysis, B7-H6 was not detected on EVs, neither isolated from wildtype nor B7-H6 overexpressing cells. However, around 10% of EVs purified from BAG6-overexpressing cells were positive for BAG6, which was not detectable on EVs from wildtype or B7-H6-overexpressing cells (Figure 2B). This is in line with previous results, suggesting that vesicle-associated BAG6 is stress-inducible and rarely detectable on EVs purified from cells under non-stressed conditions [27,29]. The average diameter of the vesicles was between 55 nm and 60 nm, corresponding to small EVs(data not shown).

In summary, the results suggest that B7-H6 is predominantly released as a soluble molecule, e.g., via shedding rather than in association with vesicles. In line, the serum level of soluble B7-H6 in patients with gastrointestinal tumors was identified as a predictive marker for a poor prognosis [23]. However, the role of vesicle-associated B7-H6 in patients is still under debate, but data on NK cell regulation of EV-B7-H6 are not available [30].

**Figure 3:**
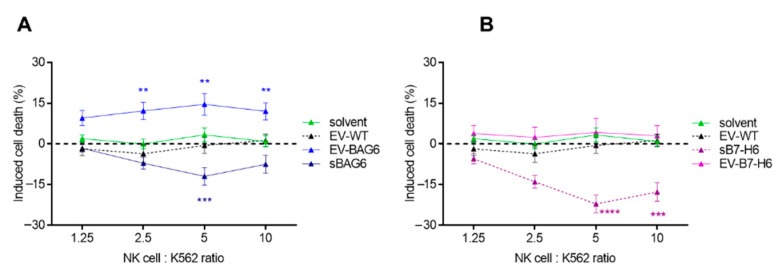
Killing response of natural killer (NK) cells after pretreatment with EVs or soluble proteins. NK cells were either (**A**) pretreated with EV-BAG6 and sBAG6 or (**B**) with EV-B7-H6 and sB7-H6 for 24 h before killing assays using K562 were performed. The killing of untreated NK cells was subtracted from treated samples, and induced killing is displayed. Data are the mean of four to eleven experiments ± SEM. Statistical significance was calculated using 2-way ANOVA and Tukey’s multiple comparison analyses, ** *p* < 0.01, *** *p* < 0.001, **** *p* < 0.0001.

Therefore, both vesicles and the soluble proteins were purified from the supernatant of transfected cells, and the impact of these fractions on NK cell activity was analyzed. The purified ligands and vesicles were preincubated with primary NK cells from healthy donors for 24 h. The EVs or the recombinant proteins did not affect the proliferation of NK cells within this experimental setting. NK cells were then cocultivated with K562 (target cells) to measure NK cell-mediated tumor cell killing. In line with previous data, we observed an increased killing response of ~15% when the NK cells were incubated with EV-BAG6, whereas NK cell-killing was inhibited by the soluble protein (Figure 3A). Activation of NK cell activity in response to preincubation with EV-B7-H6 was not observed. However, the soluble protein isolated from the cell supernatant mediated a significant decrease of NK cell cytotoxicity (Figure 3B).

**Figure 4:**
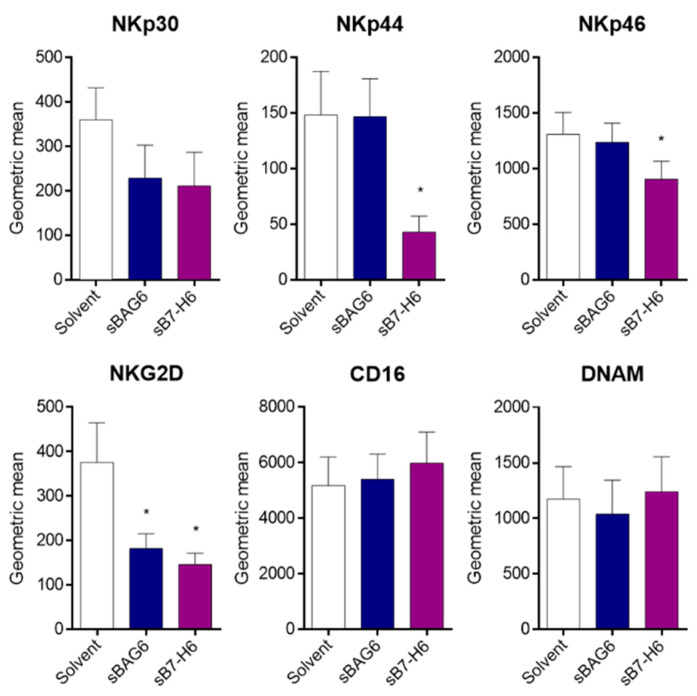
Reduced NK cell marker expression after treatment with soluble protein. NK cells were treated with sBAG6 or sB7-H6 for 24 h before cells were collected and NK cell marker expression was measured. Data are the mean of four to eight experiments ± SEM. Statistical significance was calculated using Wilcoxon test, * *p* < 0.05.

Flow cytometry analysis of NK cells showed that the surface expression of the main cytotoxicity receptors NKp30, NKp44, NKp46, and NKG2D was diminished upon treatment with the soluble NKp30 ligands (Figure 4). The percentage of positive cells decreased accordingly except of the highly expressed receptor NKp46, which was still detectable on all NK cells (Supplementary Figure S1). The decreased expression was in line with their reduced cytotoxicity and resembled partly the constricted phenotype of peripheral NK cells of tumor patients [2]. Different from the coordinated downregulation of NCRs and NKG2D, other cytotoxicity receptors, such as CD16 and DNAM, which are also downregulated in tumor patients, remained unaffected.

**Figure 5:**
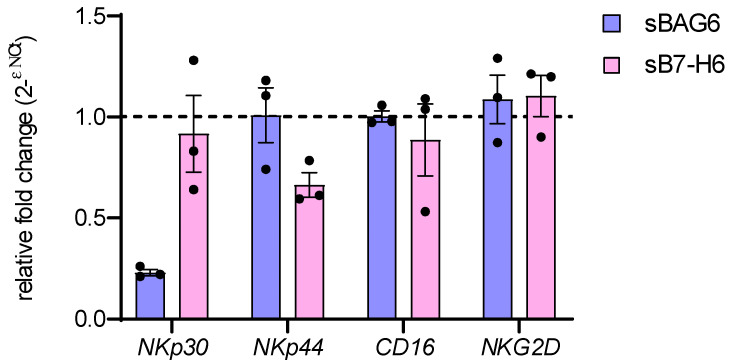
*NKp30* mRNA expression decreases upon treatment with soluble BAG6. NK cells were treated for 24 h with soluble BAG6 (sBAG6) or B7H6 (sB7H6). Fold change of mRNA expression of *NKp30*, *NKp44*, *CD16*, and *NKG2D* was analyzed by RT-qPCR and normalized to *GAPDH* and *L27* mRNA (2^ΔΔCt^). Three biological replicates (donors) were analyzed.

The correlation of high sB7-H6 patient serum level and the reduction of NK cell functions upon recognition of B7-H6-expressing tumor target cells in neuroblastoma was attributed to masking the surface receptor by the soluble ligand (25). In contrast, sBAG6 serum levels were specifically associated with the downregulation of *NKp30* mRNA transcription in these patients [25]. We, therefore, used our experimental system to directly test to what extent *NKp30* mRNA was regulated by soluble NKp30 ligands (Figure 5). Quantitative real-time RT-PCR showed significant inhibition of *NKp30* mRNA expression in response to sBAG6, whereas treatment with sB7-H6 did not affect *NKp30* mRNA level. Interestingly, this was a specific effect on *NKp30* mRNA and not a general effect on receptor mRNA expression, since mRNA levels of *NKp44*, *CD16*, and *NKG2D* remained unaffected (Figure 5). These data suggest that sB7-H6 and sBAG6 inhibitory activities on receptor expression depend on distinct cellular mechanisms, which might be considered concerning for novel immunotherapeutic strategies aiming at restoring NKp30 function.

The immunosuppressive activity of sBAG6 versus vesicle-associated BAG6 was already reported and, in addition, confirmed in a clinical study with EVs of autologous dendritic cells [31]. These BAG6-positive EVs were able to significantly improve the NKp30-mediated activity of NK cells in non-small cell lung cancer (NSCLC) patients with low NKp30 expression. The efficacy of NK cell activation could also be correlated with the BAG6 expression on the immune-stimulating EVs and the progression-free survival of the patients [31]. The inhibition of NK cells through B7-H6 was demonstrated, but it was not analyzed whether the effect was mediated by a soluble or vesicle-associated protein [14,30]. This study shows that B7-H6 is not recruited into EVs, as shown for BAG6. Recently, the protein motif PS/TAP was identified as an “EV-targeting sequence” that interacts with the ESCRT complex (endosomal sorting complex required for transport) [29,32]. As expected, this motif is present in the BAG6 protein but absent in B7-H6. Another soluble ligand of NKp30, which is released by tumor cells, is galectin-3 [33]. This protein also harbors a PS/TAP motif and is recruited into extracellular vesicles; however, it is not exposed on the vesicle surface [32], suggesting that vesicle-associated galectin-3 does not interact with NKp30 expressed on NK cells. BAG6 and B7-H6, in their soluble forms, both inhibit NK cell function but do so by distinct mechanisms, since only sBAG6 affects NKp30 expression on the transcriptional level. Further research on the soluble and vesicle-mediated regulation of NK cells via the NKp30 ligand/NKp30 axis and the elucidation of underlying molecular mechanisms is of importance in view of the clinical relevance and potential therapeutic approaches.

## 3. Materials and Methods

### 3.1. Transient Transfection with BAG6 or B7-H6-Expressing Vectors

pcDNA3.1 containing full-length B7-H6 was kindly provided by A. Cerwenka and was recloned into a pcDNA3.1/*myc*-His A vector. HEK293 cells were cultivated in DMEM (Gibco^TM^, Life Technologies, Carlsbad, CA, USA) with 10% fetal calf serum (FCS) (Gibco^TM^) and were transfected with vectors containing the sequence for either BAG6 (https://doi.org/10.1016/j.immuni.2007.10.010) or B7-H6 using the calcium phosphate precipitation method (doi:10.21769/BioProtoc.86). After 48 h post-transfection, cells and supernatant were harvested for cell and EV collection.

### 3.2. EV Isolation and Purification of His-tagged BAG6/B7-H6

Cells and supernatant were collected and centrifuged at 300× *g* for 5 min at 4 °C. The supernatant was subsequently centrifuged at 2000× *g* for 15 min, 10,000× *g* for 1 h, and then pelleted at 100,000× *g* for 90 min in an Optima XPN-80 ultracentrifuge (Beckman Coulter, Krefeld, Germany) using an SW32Ti swing-out rotor. The supernatant was collected for the isolation of His-tagged soluble BAG6 and B7-H6 proteins. The EV pellet was resuspended in 1x Hank’s balanced salt solution and centrifuged at 100,000× *g* for 100 min in an Optima MAX-XP (Beckman Coulter) centrifuge using a TLA-45 fixed angle rotor. The pellet was resuspended in 50–100 µL 1× HBSS and stored at −20 °C until the start of the experiment. Soluble proteins were purified using crosslinked nickel–nitriloacetic acid (Ni–NTA) agarose following the protocol of the company (Qiagen, Hilden, Germany). The protein concentration was determined by Coomassie gel staining and spectrophotometry.

### 3.3. EV Characterization by NanoFCM and Flow Cytometry-Bead Assay

For nano flow cytometry of isolated EVs, a flow nano analyzer (NanoFCM Co. Ltd., Nottingham, UK) equipped with a 488 nm laser was calibrated using 200 nm polystyrene beads (NanoFCM Co. Ltd.), with a defined concentration of 2.08 x 10^8^ particles/mL. In addition, monodisperse silica beads (NanoFCM Co. Ltd.) of four different sizes (68 nm, 91 nm, 113 nm, 155 nm) were used as a size reference standard. Freshly filtered (0.1 µm) 1× phosphate buffered saline (PBS) was analyzed as background signal and subtracted from the other measurements. Each distribution histogram or dot-plot was derived either from data collected for 1 min (particle concentration) or after detecting approximately 4000 events (fluorescent measurements), with a sample pressure of 1.0 kPa. The EV samples were diluted with filtered (0.1 µm) 1× PBS, resulting in a particle count in the optimum range of 2500–12,000 events. Particle concentration and size distribution were calculated using the NanoFCM software (NF Profession V1.08, NanoFCM Co. Ltd.). For immunofluorescent staining, the following antibodies were used: FITC-conjugated mouse antihuman CD9 antibody (clone HI9a, BioLegend, Koblenz, Germany), FITC-conjugated mouse antihuman CD81 antibody (clone TAPA-1, BioLegend), PE-conjugated mouse antihuman CD63 antibody (clone H5C6, BioLegend), PE-conjugated antihuman B7H6 (clone 875001, R&D systems, Minneapolis, MN, USA), and antihuman BAG6 (Schuldner et al. 2019), with secondary FITC-conjugated goat antimouse antibody (clone poly4053, BioLegend). As isotype controls, FITC-conjugated mouse IgG1, κ (clone MOCP-21, BioLegend) and PE-conjugated mouse IgG2a, κ (clone MOPC-1739, BioLegend) were used. In total, 0.2–2 ng/µL of each antibody in 100 µL 1× PBS was used. For the characterization of EVs via flow cytometry bead assay, EVs were incubated with Polybead^®^ Microspheres 4.50 µm (Polysciences Europe, GmbH, Hirschberg an der Bergstrasse, Germany) overnight at 4 °C. EVs were blocked with an equal volume of 2% bovine serum albumin (BSA) (Carl Roth, Karlsruhe, Germany) for 1 h at room temperature (RT). The EV-bead mixture was divided for the individual antibody staining. The EVs were then incubated with the following antibodies: anti-Hsp70 (clone C92F3A-5, Enzo LifeSciences, Lausen, Germany), anti-MHCI (G46-2.6, BD Biosciences, Heidelberg, Germany), anti-CD9 (clone HI9a, BioLegend), anti-CD63 (clone Ts63, Thermo Fisher Scientific, Waltham, MA, USA), and anti-BAG6. As isotype control, LEAF™ purified mouse IgG1, κ Isotype Ctrl (clone MOCP-21, BioLegend), was used. Then, samples were incubated with secondary FITC-conjugated goat antimouse antibody for 30 min on ice. Samples were washed in FACS buffer and were measured by flow cytometry using a FACS Canto II cytometer. Samples were analyzed by FlowJo (version 10.6.1, BD Bioscience).

### 3.4. Western Blot Analysis

Cells were lysed with 1× radioimmunoprecipitation assay (RIPA) buffer, and EVs, resuspended in 1× PBS, were lysed in 5× RIPA buffer; the protein concentration was determined by Pierce^TM^ BCA protein assay (Thermo Fisher Scientific). A 4× SDS sample buffer was added, and samples were run on a 10% SDS protein gel. Proteins were transferred to a nitrocellulose membrane (GE Healthcare, Freiburg, Germany), and membranes were blocked with 5% (*w/v*) milk powder (Carl Roth) in Tris-phosphate-buffered saline supplemented with 0.05% Tween (TBS-T) for 1 h. Subsequently, membranes were probed using the following antibodies: anti-Calnexin (clone AF18, Santa Cruz Biotechnology, Heidelberg, Germany), anti-Flotillin-1 (clone 18, BD Biosciences), anti-GAPDH (G9545, Sigma-Aldrich, Munich, Germany), anti-BAG6, and anti-B7-H6 (ab121794, Abcam, Cambridge, UK). Detection was performed with horseradish peroxidase-conjugated secondary antibodies (DAKO, Hamburg, Germany) using Amersham ECL Plus (GE Healthcare).

### 3.5. NK Cell Isolation, Cultivation, and Killing Assay

Peripheral blood mononuclear cells (PBMCs) were purified by ficoll density centrifugation using LSM1077 (Sigma-Aldrich). NK cells were isolated using a negative selection NK cell isolation kit (Miltenyi Biotec, Bergisch Gladbach, Germany). Isolated NK cells were cultured in IMDM medium (Gibco^TM^) with 10% FCS, 1% P/S, and 10 U/mL IL-2 and rested at 37 °C overnight. NK cells were pretreated with 100 µg/mL EVs or soluble protein for 24 h before killing assays were performed. For killing assays, K562 target cells were stained with 5 µM CellTracker^TM^ Violet BMQC (Life Technologies) fluorescent dye in serum-free medium at 37 °C for 45 min. Then, FCS was added to the cells at a final concentration of 20%. Cells were resuspended in fresh medium and seeded accordingly in IMDM medium with 10% FCS and 1% P/S in U-shaped, 96-well plates. NK cells were added to the target cells in ratios ranging from 1.25:1 to 10:1. The cells were cocultured for 3 h before they were harvested and centrifuged at 300× *g* for 5 min. The supernatant was discarded, and the cells were resuspended in 200 µL PBS before they were stained with 100 ng/200 µL 7-AAD (BioLegend). Cell death was measured by flow cytometry using a FACS Canto II cytometer (BD Bioscience) and analyzed by FACS Diva software. The killing of untreated NK cells was subtracted from the treated samples and induced killing was displayed.

### 3.6. NK Cell Receptor Surface Marker Expression

NK cells were treated with soluble protein or solvent (PBS) for 24 h. Then, the cells were collected and centrifuged at 300× *g* for 5 min at RT. Cells were resuspended in PBS and incubated with the following antibodies: CD3-V500 (clone UCHT1, BD Biosciences), CD16-APC-Cy7 (clone 3G8, BioLegend), CD56-PerCP-Cy5.5 (clone HCD56, BioLegend), NKG2D-FITC (clone 1D11, BioLegend), NKp44-AF647 (clone P44-8, BioLegend), NKp30-BV421 (clone p30-15, BD Biosciences), NKp46-PE (clone9E2, BioLegend), and DNAM-PE-Cy7 (TX42.1, BioLegend). The IgGk controls coupled with the corresponding fluorophores were purchased from BioLegend and BD Biosciences. The cells were incubated for 30 min at RT and washed in PBS, and marker expression was measured on a FACS Canto II cytometer. The background signal of the IgG controls was subtracted, and the signal of the geometric mean was displayed.

### 3.7. Quantitative RT-PCR

RNA was isolated using TriFast™ (VWR Peqlab, Darmstadt, Germany) in combination with NucleoSpin RNA Kit (Macherey–Nagel, Düren, Germany). Briefly, cell pellets were lysed in 500 µL TriFast, and 100 µL chloroform was added. The samples were mixed and centrifuged at 13,300× *g* for 15 min at 4 °C. The aqueous phase was collected, and 100% ethanol was added in a 1:1 ratio. The samples were then desalted, washed, and eluted according to the instructions of the kit. mRNA was transcribed into cDNA using the RevertAid First Strand cDNA Synthesis Kit (Thermo Fisher Scientific), according to the manufacturer’s instructions. The following primers were used: NKp30 total forward 5′-AAGTGATGTGTGAGTCCCGT-3′ and reverse 5′-CACTACTTGTAGCCAGGCCT-3′, NKp44 forward 5′-ACGAGAAGAAAGGCTGGTGT-3′ and reverse 5′-AATCGAGAGGTCCAAGCCAT-3′, CD16 forward 5′-GCCCATGATCTTCAAGCAGG-3′ and reverse 5′-TTGCTTTGCTGTGAGGGAAC-3′, NKG2D forward 5′-ATGGATCTTGGCAGTGGGAA-3′ and reverse 5′-GAGTGCACAGTCTCCCTTCT-3′, GAPDH forward 5′-TGCACCACCAACTGCTTAGC-3′ and reverse 5′-GGCATGGACTGTGGTCATGAG-3′, L27 forward 5′-AAAGCTGTCATCGTGAAGAAC-3′, and 5′-GCTGTCACTTTGCGGGGGTAG-3′. The qPCR was carried out using ABsolute QPCR Mix SYBR Green (Thermo Fisher Scientific) on an Mx3000P (Agilent Technologies, Santa Clara, CA, USA) thermocycler. The relative expression level was calculated by the 2-^∆∆ct^ method, in which the two housekeeping genes GAPDH and L27 were used for normalization.

### 3.8. Statistical Analysis 

Statistical significance was calculated using Prism v6.0 software (GraphPad, La Jolla, CA, USA). One-way and two-way ANOVA with Tukey’s multiple comparisons tests were applied as indicated in the figure legends.

### 3.9. Ethics Statement

The study was conducted according to the guidelines of the Declaration of Helsinki, and approved by the Ethics Committee of the Department of Medicine, Philipps University of Marburg (Votum Az 13/15, date of approval 7 December 2017).

## Data Availability

The data presented in this study are contained within the article or supplementary material.

## Supplementary Materials

The following is available online at https://www.mdpi.com/1422-0067/22/4/2189/s1. Figure S1: Percentage NK cell marker expression after treatment with soluble protein.
