# Supplementary material for: Secreted Ligands of the NK Cell Receptor NKp30: B7-H6 Is in Contrast to BAG6 Only Marginally Released via Extracellular Vesicles
Source: Int J Mol Sci. 2021 Feb 22;22(4):2189. doi: 10.3390/ijms22042189 (PMC7926927; doi:10.3390/ijms22042189)
Supplement: Supplementary file 1 [file ijms-22-02189-s001.pdf]

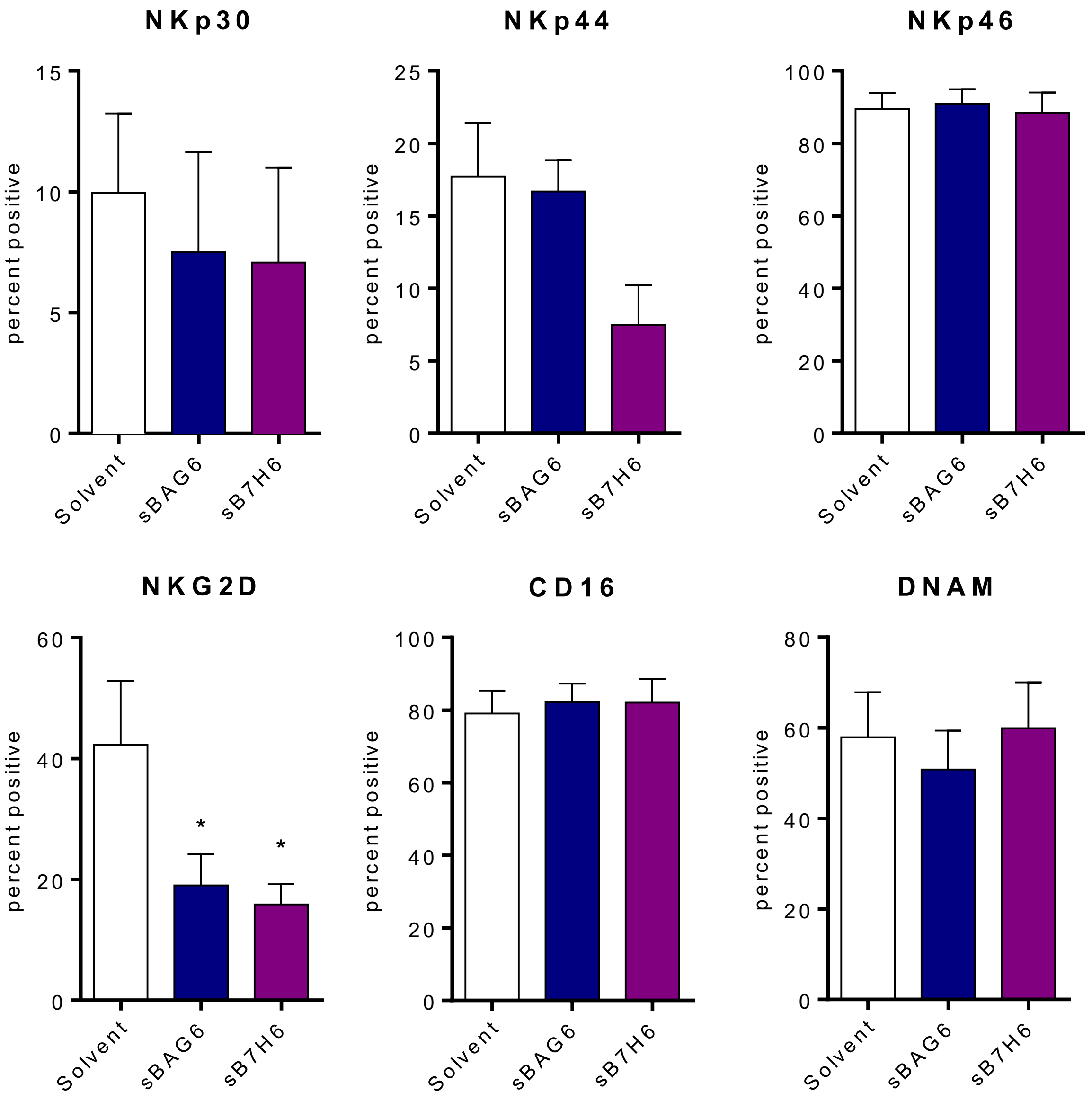

Figure S1. Percentage NK cell marker expression after treatment with soluble protein. NK cells were treated with sBAG6 or sB7-H6 for 24 h before cells were collected and NK cell marker expression was measured by flow cytometry. Data are the mean of four to eight experiments ± SEM. Statistical significance was calculated using Wilcoxon test, *$p < 0.05$
